# Synaptic-like plasticity in 2D nanofluidic memristor from competitive bicationic transport

**DOI:** 10.1126/sciadv.adr1531

**Published:** 2024-11-06

**Authors:** Yechan Noh, Alex Smolyanitsky

**Affiliations:** ^1^ Department of Physics, University of Colorado Boulder, Boulder, CO 80309, USA.; ^2^ Applied Chemicals and Materials Division, National Institute of Standards and Technology, Boulder, CO 80305, USA.; ^3^ Department of Materials Science and Engineering, University of California, Berkeley, Berkeley, CA 94720, USA.

## Abstract

Synaptic plasticity, the dynamic tuning of signal transmission strength between neurons, serves as a fundamental basis for memory and learning in biological organisms. This adaptive nature of synapses is considered one of the key features contributing to the superior energy efficiency of the brain. Here, we use molecular dynamics simulations to demonstrate synaptic-like plasticity in a subnanoporous two-dimensional membrane. We show that a train of voltage spikes dynamically modifies the membrane’s ionic permeability in a process involving competitive bicationic transport. This process is shown to be repeatable after a given resting period. Because of a combination of subnanometer pore size and the atomic thinness of the membrane, this system exhibits energy dissipation of 0.1 to 100 aJ per voltage spike, which is several orders of magnitude lower than 0.1 to 10 fJ per spike in the human synapse. We reveal the underlying physical mechanisms at molecular detail and investigate the local energetics underlying this apparent synaptic-like behavior.

## Teaser

Competitive sieving of aqueous salt mixture through subnanoporous 2D membranes enables dynamic potentiation of ion transport.

## INTRODUCTION

Synapses are junctions that enable chemo-electrical signaling between neurons. In a typical synapse, the signal transmission strength is dynamically modulated in response to previous neural activity, a feature referred to as synaptic plasticity (*1*, *2*). This adaptive alteration of synaptic strength plays a fundamental role in memory and learning functions in living organisms. Moreover, it enables biological neural networks to concurrently perform both processing and storage of information in a sparse manner, a feature believed to be central to their superior energy efficiency. Inspired by these biological functionalities, artificial electrical elements with synaptic-like plasticity have been studied extensively (*3*–*8*), aimed at building analog artificial neural networks with substantially enhanced energy efficiency compared to emulations based on the von Neumann computing architecture.

Memristors (*9*) comprise an area of extensive research due to their potential promise as artificial synaptic elements for neuromorphic computing. Physically, a memristor is an electrical conductor capable of modulating its conductivity in response to previous voltage inputs and maintaining the modulated state without a continuous source of power. This internal gating enables memristor networks to perform information processing and storage simultaneously. Over the past decade, solid-state neuromorphic chips featuring memristor networks have been demonstrated to perform analog machine learning tasks at a fraction of the energy cost of their von Neumann counterparts (*5*, *10*–*12*). More recently, there has been a spike of interest in nanofluidic memristors to directly mimic biological neural networks (*13*–*25*), with two recent works notably reporting long-term memory effects along with basic Hebbian learning (*18*) as well as operating voltage comparable to that of biological synapses and yielding subpicojoule energy consumption per spike (*17*). In nanofluidic memristors, aqueous ions serve as the charge carriers instead of electrons, in resemblance to the human brain. One noteworthy difference between electrons and ions as charge carriers is the rich diversity of the latter, which can coexist within a given system. In particular, the competitive interplay between the ionic species leads to interesting phenomena, including ion sieving (*26*) and memristive ion transport (*22*). It is well known that biological systems readily harness the diversity of ion species for their functions, as most notably exemplified by the generation of action potentials in neurons (*27*). Therefore, exploring ways to harness ionic diversity in artificial nanofluidic systems to achieve neuromorphic functions may represent a crucial research direction in nanofluidics.

Among fluidic ion conductors, nanoporous two-dimensional (2D) membranes represent a class of materials with high energy efficiency of ion transport. The primary reason for this efficiency is the atomic thinness, which, combined with subnanometer pore dimensions, enables relatively low, highly localized, and ion-selective permeation barriers, as described earlier (*26*, *28*–*30*). Unsurprisingly, this class of materials has been considered for a range of applications, including water desalination (*31*–*34*), molecular separation (*31*, *35*, *36*), and osmotic energy harvesting (*37*, *38*). Combined with high permeation selectivity and the prospect of high-density pore array fabrication (*39*–*41*), subnanoporous 2D membranes appear to be excellent candidates for artificial synaptic devices. Achieving reliable memory functionality, however, is neither trivial nor necessarily intuitive. Here, we demonstrate synaptic-like plasticity of aqueous ion transport through a subnanoporous 2D membrane. We demonstrate that it arises from the adsorption/desorption and transport of two cation species with markedly different ion-pore affinities. Finally, we provide a comprehensive molecular-level insight into the underlying mechanisms.

## RESULTS AND DISCUSSION

**Fig. 1.**
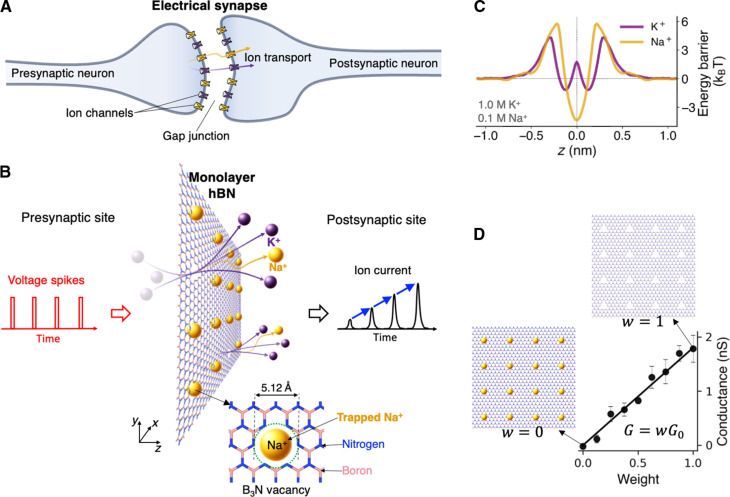
Sketch of the system and characteristics of ion transport across a subnanoporous hBN monolayer. (**A**) Illustration of biological electrical synapse and (**B**) 2D porous membrane involving bicationic ion transport driven by spiking voltage, which causes desorption of Na^+^ ions from the pores and activating K^+^ transport. (**C**) Energy profile for K^+^ and Na^+^ ions along the transport coordinate for a binary salt mixture containing 1.0 M KCl and 0.1 M NaCl. (**D**) Membrane conductance versus weight at 1.0 M K^+^. The insets in (C) show Na^+^ trapping states at the corresponding weight values.

We used all-atom molecular dynamics (MD) simulations to investigate dynamic ion transport across a 2D porous membrane under a sequence of rectangular voltage pulses. Figure 1A shows a sketch of ion transport in a biological electrical synapse, where ions are transported between the ion channels across the gap junction between the presynaptic neuron and the postsynaptic neuron. The plasticity of such synapses typically arises from the dynamic changes in conductance of the voltage-gated channels, as well as the gap region (*42*, *43*). Here, we considered a subnanoporous 2D membrane mimicking a simplified artificial synapse. Figure 1B illustrates bicationic (Na^+^ and K^+^) ion transport through an array of subnanometer pores hosted by a 2D membrane. In this case, the membrane material is a hexagonal boron nitride (hBN) monolayer with a total of 16 regularly spaced B_3_N triangular multivacancy pores within an area of approximately 7 nm × 7 nm. The B_3_N pore is a defect commonly found in monolayer hBN (*44*) and its effects on wettability (*45*), water slippage (*46*), as well as ion trapping and mechanosensitive ion transport (*47*) have been studied. The membrane is suspended in the middle of a simulation box and immersed in a binary mixture of water-dissociated 1.0 M KCl and 0.1 M NaCl, unless the concentrations are stated otherwise. Dynamic transport response in this system is initiated by a sequence of rectangular electric field pulses externally applied in the *Z* direction, as shown in Fig. 1B. Further details on the simulation procedures can be found in Methods.

The permeation properties of B_3_N vacancies in hBN are worth introducing first. These electrically neutral subnanometer pores feature dipolar electrostatics with negatively charged dipole components located at the edge nitrogen atoms, enabling selective transport. More specifically, anions are outright rejected, while K^+^ ions permeate relatively rapidly and Na^+^ ions hardly permeate as they become stably trapped in the pores (*47*). Figure 1C shows the corresponding free energy landscapes in the form of potentials of mean force (PMF) along the transport coordinate (*Z*) for both ion species. For Na^+^, the energy profile features a barrier of ≈10*k*_B_*T* at *Z* = 0 (*k*_B_ is the Boltzmann constant and *T* = 300 K is the system temperature). The K^+^ ions, however, experience a much shallower energy well, ≈5.5*k*_B_*T* deep, resulting in relatively weak trapping of K^+^ ions. As described earlier (*26*, *28*, *29*), the rate of ion transport through these barrier-limited pores is of the Arrhenius type, i.e., $I\propto e^{-\frac{\Delta E}{k_{B}T}}$, where Δ*E* is the rate-setting peak-to-peak free energy barrier. Unsurprisingly, given an energy barrier difference of ≈4.5*k*_B_*T*, the rate of K^+^ permeation is nearly two orders of magnitude higher than that of Na^+^ in a single-salt scenario. Following from the same argument, Na^+^ ions spend substantially more time trapped inside the pores, compared to K^+^, which overall is similar to 18-crown-6 ether pores in graphene, except that the ion-pore affinity is reversed for the same cation pair (*26*, *28*). Given that a pore of this size becomes impermeable when a cation is trapped inside, the number of available conductive paths is the total number of pores unoccupied by Na^+^. The effective membrane conductance for a salt mixture can thus be written as a simple linearly weighted function proposed earlier (*22*), directly evaluated in Fig. 1D

$$G={\mathrm{wG}}_{0}$$ (1)

where *G*_0_ is the conductance of a membrane completely deoccupied by Na^+^. The corresponding weight *w* is the time-dependent fraction of empty pores: *w*(*t*) = 1 − *N*(*t*)/*N*_tot_, where *N*(*t*) is the number of pores “plugged” by Na^+^ and *N*_tot_ is the total number of pores in the array.

**Fig. 2.**
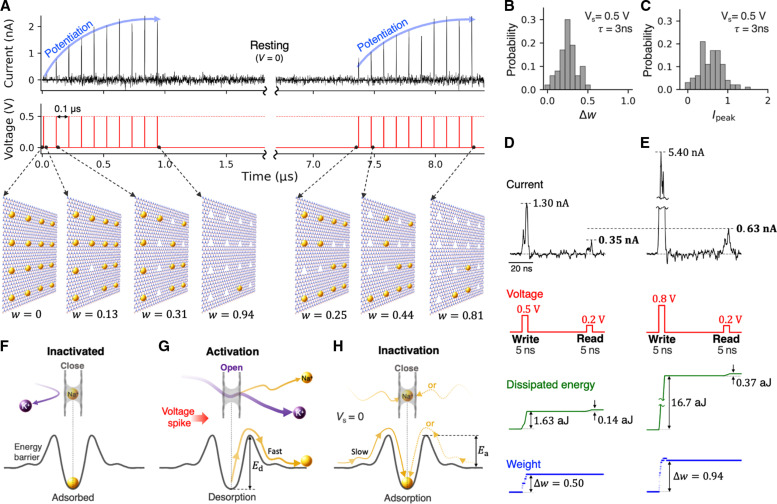
Synaptic-like plasticity in ion transport across an hBN monolayer membrane. (**A**) Ion current potentiation by rectangular voltage pulses. In the resting cycle, the system is unbiased. The subfigures illustrate trapped Na^+^ ions at selected times. The probability distribution of (**B**) pulse-induced weight increments and (**C**) current peaks obtained from 100 independent simulations with a 0.5-V voltage pulse of τ = 3 ns. Writing and reading operations using a write pulse of (**D**) 0.5 V and (**E**) 0.8 V, followed by a 0.2-V read pulse. All read and write pulses were 5 ns long. The interval between writing and reading operations is 50 ns. Mechanisms associated with the synaptic-like plasticity: (**F**) Inactivated state: the trapped Na^+^ blocks K^+^ transport. (**G**) Activation or learning process: bias-induced desorption of Na^+^. (**H**) Inactivation or forgetting process: readsorption of Na^+^ ions.

The results for synaptic-like plasticity exhibited by the aqueous ion transport through an array of B_3_N vacancies in a 2D hBN monolayer under a pulsed bias are shown in Fig. 2. In the context of this work, plasticity refers to the dynamic alteration of the membrane’s ionic permeability in response to voltage bias history. We first applied ten 3-ns-long, 0.5-V voltage pulses with an interval of 0.1 μs (results obtained for different intervals can be found in fig. S1). Before the first voltage pulse, none of the pores were activated, i.e., all 16 pores were occupied by the trapped Na^+^ ions. The pore activation dynamics as a function of subsequent voltage pulses is shown in Fig. 2A, with the first pulse activating two pores and the second pulse activating three more pores, corresponding to *w* = 2/16 = 0.125 and *w* = 5/16 = 0.3125, respectively—and so forth. At a finite temperature, the individual Δ*w* increments are of course stochastic and thus the analytical discussions provided later in the text correspond to the statistically significant Δ*w* values, i.e., those obtainable from repeated identical pulse trains applied to a system initially at *w* = 0. The distributions of weight change and peak current (*I*_peak_) resulting from a 3-ns-long 0.5-V pulse are shown in Fig. 2, B and C, respectively. The corresponding average weight change is 0.256 with an SD of 0.108 for a membrane featuring 16 pores. This type of stochasticity introduces a degree of inherent randomness, which results in natural diversity in conductance switching. In principle, intrinsic stochasticity is commonly found in solid-state memristors (*10*) and the human brain (*4*). Specifically for the presented system, the degree of randomness should decrease with increasing array size. As successive voltage pulses are applied, these spikes expectedly exhibit an increasing trend with a clear asymptote corresponding to the maximum weight *w* = 1 (at the end of the first 10-pulse sequence, the value of *w* increased to 0.94, corresponding to only one trapped Na^+^ ion). The information about the previous history of voltage pulses is stored in the form of cumulatively added deoccupancy of Na^+^. This gradual potentiation of ion permeability arises due to the competitive transport between K^+^ and Na^+^, and thus, the potentiation effects do not take place with a monocationic electrolyte (see fig. S2). As expected for a system symmetric with respect to the membrane, the potentiation occurs regardless of bias reversal, resulting in bidirectional memristive behavior described in fig. S3. Once the pulse train stops, the system undergoes resting, during which the Na^+^ ions are slowly re-trapped, volatilizing the previously gained memory. After approximately 6 μs of rest, 11 additional Na^+^ ions are re-trapped, leaving only four pores empty and corresponding to *w* = 4/16 = 0.25. After that, the system underwent another learning cycle with the same sequence of voltage pulses, exhibiting ion current potentiation similar to that observed in the first cycle, except with a different initial value of *w*. As demonstrated, the conductance state of the device is switchable by voltage pulses a few nanoseconds in duration, resulting in a switching resolution at the scale of order 0.1 GHz. The relaxation time of a few microseconds, on the other hand, corresponds to the effective memory retention time, tunable by the local association barriers, as well as the ion concentrations, as discussed in greater detail further in the text. These timing ranges may therefore be of specific interest for implementing devices that aim to combine gigahertz-scale state switching with megahertz-scale state retention.

Given the discussion above, the high-magnitude component of the bias pulse can be viewed as the write operation performed on a membrane, while a measurement of the ion current during the lower-magnitude pulse is the read operation aimed at probing the membrane’s ionic permeability. For writing, we used 5-ns-long pulses of relatively high magnitude (0.5 V and higher), which enable rapid removal of some of the Na^+^ ions from the pores. For reading, pulses of lower magnitude (0.2 V) were used, allowing to merely probe the membrane permeability in its Na^+^-occupancy state without modifying the latter. Figure 2 (D and E) shows the corresponding results for write pulses of 0.5 and 0.8 V, followed by a read pulse after 50 ns. As expected, permeability potentiation is stronger with a write pulse of higher magnitude: Δ*w* = 0.50 and Δ*w* = 0.94 for 0.5 and 0.8 V pulse, respectively. The corresponding dissipated energy expenditure is quite low. We define the energy dissipated in a given pulse as $\Delta E=\int_{0}^{\tau }I\left(t\right)V_{s}\mathrm{dt}$, where *I*(*t*) is the ion current and *V*_s_ is the pulse height, integrated for the pulse duration τ. As shown in Fig. 2, (D and E), the energy dissipation per voltage spike in this 2D nanofluidic memristor is on the attojoule scale, attributed to the relatively low ion currents and the nanosecond-scale pulse. These energy estimates are several orders of magnitude lower than those of human synapses, which have an energy expenditure of roughly 0.1 to 10 fJ per synaptic event (*6*, *8*, *48*). Note that the attojoule-scale dissipation estimate given above should only be viewed as an idealized lower bound. In a more realistic scenario, especially given the current state-of-the art fabrication techniques, parasitic effects in the form of ion current leakage and other sources would certainly increase dissipative losses. At the same time, this lower bound may serve as a suitable objective in terms of device design and fabrication.

The main mechanism underlying the simple potentiating-forgetting cycle described above is dynamic adsorption/desorption of Na^+^ ions by the pores, with inactivated and activated states of pores as sketched in Fig. 2, F and G, respectively. When a write pulse is applied, a significant probability of Na^+^ desorption arises, as set by the write-pulse voltage peak and the desorption barrier *E*_d_ (see Fig. 2G for the definition). Upon forgetting, adsorption occurs with a considerably lower probability, as set by the adsorption barrier *E*_a_ (see Fig. 2H) and the low concentration of Na^+^ ions. In particular, to achieve low memory volatility (i.e., in the form of a long forgetting time in Fig. 2A), a combination of relatively high *E*_a_ and sufficiently low Na^+^ concentration is essential, because the corresponding adsorption rate that describes the spontaneous “forgetting” process is $\propto c_{{\text{Na}}^{+}}\text{exp}\left(-\frac{E_{a}}{k_{B}T}\right)$, where *c*_Na_^+^ is the sodium concentration.

A simple analytical model describing the entire process presented above is possible. For an individual pore, the effective desorption rate in the presence of external bias is given by $r_{d}=f_{d}\text{cosh}\left(\frac{q\phi }{2k_{B}T}\right)\text{exp}\left(-\frac{E_{d}}{k_{B}T}\right)$, where *f*_d_ is the attempt frequency associated with thermal fluctuations, ϕ ∝ *V*(*t*) is the bias-induced local shift of the ion’s electrostatic potential, and *q* is the electric charge of the ion. As mentioned earlier, the concentration-dependent adsorption rate is $r_{a}=\kappa c_{{\text{Na}}^{+}}\text{exp}\left(-\frac{E_{a}}{k_{B}T}\right)$ (see Fig. 2H for the definition of *E*_a_), where *c*_Na_^+^ is the concentration of Na^+^ ions and κ is a suitable transmission coefficient such that κ*c*_Na_^+^ is the corresponding adsorption attempt frequency. In general, *r*_a_ is bias dependent, but we posit that the dependence here is considerably weaker than for desorption. For a pore array, let us consider the system state determined by the number of Na^+^ ions trapped in the pores as a function of time, in response to a voltage pulse. We note that despite the fact that the ability to observe time delays in the form of ion currents requires at least two cation species with markedly different ion-pore affinities (*22*), the time-delayed state dynamics itself can be described in a single-cation scenario. The number of trapped Na^+^ ions *N*(*t*) satisfies a simple differential equation: $\frac{\mathrm{dN}}{\mathrm{dt}}=-r_{d}N+r_{a}\left(N_{\text{tot}}-N\right)$. For a constant bias (e.g., during a rectangular voltage pulse), the analytical solution is then $N\left(t\right)=N_{0}e^{-\left(r_{a}+r_{d}\right)t}+\frac{N_{\text{tot}}r_{a}}{r_{a}+r_{d}}\left(1-e^{-\left(r_{a}+r_{d}\right)t}\right)$, where *N*_0_ is the initial state. The corresponding weight is then given by $w=w_{0}+\left(\frac{r_{d}}{r_{a}+r_{d}}-w_{0}\right)\left(1-e^{-\left(r_{a}+r_{d}\right)t}\right)$, where *w*_0_ = 1−*N*_0_/*N*_tot_ is the initial weight. Given that *r*_d_ is an exponential function of the external bias, two distinct processes are possible, depending on the relative strengths of *r*_a_ and *r*_d_.

**Fig. 3.**
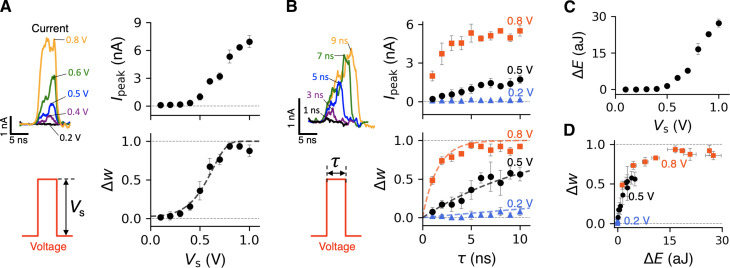
Synaptic potentiation and energy consumption during a single voltage pulse of varying magnitude and duration. Peak current and weight change for (**A**) varying *V*_s_ at fixed τ = 5 ns and (**B**) varying τ at fixed *V*_s_. (**C**) Dissipated energy as a function of *V*_s_ with a fixed τ = 5 ns (**D**) Dissipated energy versus weight change. As shown, *I*_peak_, Δ*w*, and Δ*E* are averages from four independent simulations. The error bars are the corresponding SDs.

As shown earlier, the system “learns” from a train of “writing” pulses when *r*_d_ ≫ *r*_a_ during each pulse and the interval between the pulses is insufficiently long for significant memory loss to occur. A necessary requirement here is that the bias magnitude is sufficiently high for a pulse lasting only a few nanoseconds to remove a considerable number of sodium ions from the pores. The learning process is then cumulative, i.e., the state after the *n*th pulse remembers the sum of *w* changes caused by the previous pulses, until all pores are deoccupied by Na^+^. After *n*th pulse of duration τ

$$w_{n}=w_{n-1}+\left(\frac{r_{d}}{r_{a}+r_{d}}-w_{n-1}\right)\left(1-e^{-\left(r_{a}+r_{d}\right)\tau }\right)$$ (2)

where *w*_*n*−1_ and *w_n_* are the weight before and after the pulse, respectively. At *r*_d_ ≫ *r*_a_, *w*_0_ = 0, and assuming no appreciable re-adsorption between the equation pulses, the difference equation above yields a geometric series $w_{n}=1-e^{-\left(r_{a}+r_{d}\right)\tau n}$, asymptotically convergent to unity, in accord with the results in Fig. 2A. Of potential interest, for a train of voltage pulses of constant magnitude and duration, the *w_n_*−*w*_*n*−1_ increments are statistically numerically unique. In principle, this suggests the possibility of implicitly “encoding” information about the number of pulses that preceded a given unsaturated value of *w* in the case of large pore arrays. The *r*_d_’s sensitivity to the bias magnitude is worth considering in greater detail. The results of simulated potentiation by a single 5-ns-long voltage pulse of height *V*_s_, applied to a membrane initially fully inactivated (*w* = 0), are shown in Fig. 3A. Both the current spike height and Δ*w* are marked by a threshold in the amount of ≈0.3 V, consistent with the definition of *r*_d_. Sensitive dependence on the pulse duration τ at a fixed *V*_s_ (see 0.5- and 0.8-V cases in Fig. 3B) above the threshold is also shown in Fig. 3B, causing the corresponding current peak and Δ*w* to increase rapidly when τ increases. In addition, there is reasonable agreement between Δ*w* given by Eq. 2 and the simulated data (see the dashed curves in Fig. 3, A and B). Note that the resulting energy expenditure for various values of *V*_s_ shown in Fig. 3C exhibits a rapid increase above the *V*_s_ threshold. In particular, Fig. 3D shows that energy consumption increases nonlinearly with the weight increment, naturally following the corresponding rapid increase in *I*_s_ (compare the top panels in Fig. 3, A and C).

**Fig. 4.**
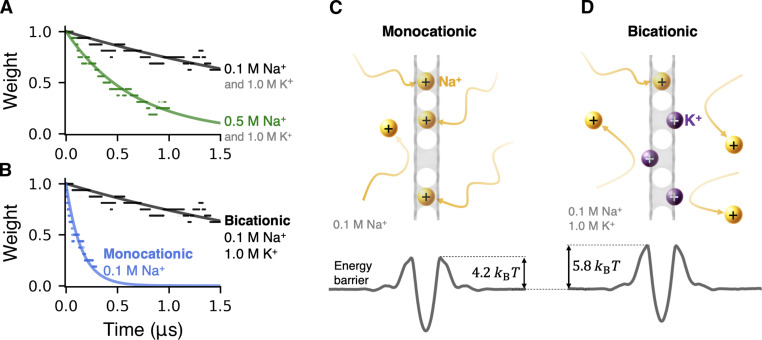
Effect of Na^+^ concentration and Na^+^-K^+^ interplay in the forgetting process. (**A**) Effect of Na^+^ concentration on weight decay. (**B**) Effect of K^+^ ions on weight decay. Sketches of the forgetting process for (**C**) monocationic electrolyte featuring only Na^+^ and (**D**) bicationic electrolyte containing Na^+^ and K^+^ at the stated ion concentrations, along with the corresponding energy profiles directly below.

The second important process that can occur is the loss of memory in the longer-term absence of external bias, which manifests as spontaneous gradual decrease in *w*. The unbiased dynamics is described identically to Eq. 2, except now *r*_a_ and *r*_d_ are more comparable

$$w=w_{0}+\left(\frac{r_{d}}{r_{a}+r_{d}}-w_{0}\right)\left(1-e^{-\left(r_{a}+r_{d}\right)t}\right)$$ (3)

where *w*_0_ > 0 is the state before the start of memory loss and *t* is the elapsed time. The decay rate is a sensitive function of *c*_Na_^+^ via *r*_a_ ∝ *c*_Na_^+^, consistent with the results in Fig. 4A, which shows spontaneous decay of *w* at several concentrations of NaCl. The final state of the membrane is also concentration dependent, because regardless of *w*_0_, at *t* → ∞, Eq. 3 yields $w=\frac{r_{d}}{r_{a}+r_{d}}=\frac{1}{\lambda c_{{\text{Na}}^{+}}+1}$, where λ is a constant—also consistent with the results in Fig. 4A. An important factor affecting the forgetting process omitted in the discussion above is the presence of K^+^ ions, which are expected to interfere with Na^+^-pore binding. As shown in Fig. 4B, the presence of K^+^ ions slows down the rate of *w* decay (also see section S2). The effects of potassium are deeper than the level of interference captured by our analytical model. Shown in Fig. 4 (C and D) is the effect of K^+^ on the barrier heights, because K^+^ ions contribute short-range steric and longer-range electrostatic cation-cation repulsion through their presence in the direct vicinity of the pores and near the membrane surfaces, respectively. These observations suggest that the concentration of the main conducting ion species can also be used as a tuning parameter for controlling the system dynamics. The possible influence of spurious defects essentially guaranteed to be present in realistic postfabricaton hBN membranes is worth noting briefly. Smaller defects (e.g., in the form of single-atom or B_2_N vacancies) are expected to be entirely impermeable to K^+^ and Na^+^ ions, because the B_3_N vacancy is essentially the smallest permeable pore for these cation species. At the same time, smaller cations, such as Li^+^ and protons, may still be able to permeate through smaller defects. Larger multivacancies, on the other hand, are not expected to trap ions, and thus, the transport contributions from those pores would likely manifest as baseline leakage current. Although fabrication of large uniform arrays of B_3_N vacancies remains a challenge, a new fabrication method reported recently shows substantial promise in achieving increased control over pore size and shape distribution in an array of multivacancies (*49*, *50*).

To summarize, we have demonstrated synaptic-like plasticity of aqueous ion transport through a subnanoporous 2D membrane made of a monolayer hBN featuring an array of B_3_N vacancies in a binary aqueous salt. The ion conductance of this nanofluidic memristor can be dynamically modulated by few-nanoseconds-long transmembrane voltage pulses, resulting in a switching speed of approximately 0.1 GHz. The resulting energy dissipation per voltage spike is very low, roughly 0.1 to 100 aJ per spike, which is several orders of magnitude lower than that of the human brain, 0.1 to 10 fJ per spike. Notably, this synaptic-like plasticity arises from the dynamic interplay between two cationic species (Na^+^ and K^+^), exemplifying an illustrative approach to leveraging ionic diversity for achieving neuromorphic functionality. The conductive state of the membrane is shown to be governed by the adsorption/desorption dynamics of Na^+^ trapped in the pores, while K^+^ ions permeate through the empty pores. We derived a set of illustrative analytical expressions associated with the apparent synaptic-like potentiation and memory volatility. Further experimentation is needed to demonstrate the predicted behaviors for various subnanoporous 2D membranes, as well as ionic permeants. It is our hope to stimulate further work aimed at a better understanding of the effects of ionic diversity, including at the system level, i.e., in the case of interconnected nanofluidic memristive elements.

## METHODS

For the MD simulations of ion transport, we used a rectangular box with dimension *L_X_* = 6.998 nm × *L_Y_* = 6.926 nm × *L_Z_* = 10 nm, periodic in all directions. The subnanoporous hBN monolayer was placed in the *XY* plane at *Z* = *L_Z_*/2 with the pore array consisting of 16 triangular B_3_N vacancies. To prevent the membrane from drifting, its edge atoms were tethered to their initial positions by spring restraints. The system was filled with aqueous salt mixtures consisting of explicitly simulated K^+^, Na^+^, and Cl^−^ ions. As the system was periodic in all directions, the time- and ensemble-averaged salt concentration on each side of the membrane was identical under zero bias. The membrane was simulated using parameters developed earlier (*51*) within the all-atom optimized potentials for liquid simulations (OPLS-AA) forcefield framework (*52*). The partial charges of the nitrogen atoms at the edges of B_3_N vacancies were set at 2/3 of their bulk values [obtained earlier from quantum-chemical calculations (*51*)], ensuring electrical neutrality of the membrane. Explicitly simulated water molecules were simulated using the TIP4P model (*53*). All of the nonbonded interactions were simulated using the OPLS-AA forcefield framework (*52*). A particle-particle–particle-mesh scheme was used to simulate the electrostatic interactions. A 1.2-nm cutoff radius was used for all short-range interactions, including short-range Coulomb interactions and the Lennard-Jones interactions. To initialize the conductive weight of the membrane to zero in all of our simulations, we placed Na^+^ ions inside the B_3_N vacancies. The system first underwent static energy minimization and then dynamic relaxation in the NPT ensemble at *T* = 300 K and *P* = 1 bar, using a 1-fs time step, with the Parrinello-Rahman barostat modifying simulation box dimensions only in the *Z* direction. Relaxed systems underwent ion transport simulations under rectangular pulses of external electric field applied in the *Z* direction, using a 2-fs time step. The corresponding pulse magnitudes were calculated as the electric field magnitude, multiplied by *L_Z_*. To obtain the ion currents, we first calculated the cumulative ionic fluxes as a function of time *N*(*t*). The flux data were recorded every 10 ps, corresponding to a 100-GHz sampling rate. The raw *N*(*t*) data were then filtered using a Chebyshev low-pass digital filter with a 200-MHz cutoff frequency. Finally, time derivatives of the filtered flux data were obtained using an eighth-order central difference method, yielding the time-dependent ionic current $I\left(t\right)=q\frac{{\mathrm{dN}}_{f}\left(t\right)}{\mathrm{dt}}$, where *N*_f_(*t*) is the filtered cumulative flux and *q* is the ionic charge. Further details can be found in the supplementary materials of our previous work (*22*). The PMF data were obtained using the weighted histogram analysis method (*54*) applied to a total of 60 umbrella samples of the ion’s position along the *Z* coordinate incremented by 0.05 nm; each umbrella sample was obtained from a 20-ns-long simulation. All MD simulations were performed using graphics processing unit (GPU)–accelerated GROMACS (*55*, *56*), and the molecular visualization tasks were carried using the OVITO software (*57*).

## Supplementary Materials

### This PDF file includes:

Sections S1 and S2 Figs. S1 to S6
