# Supplementary material for: Synaptic-like plasticity in 2D nanofluidic memristor from competitive bicationic transport
Source: Sci Adv. 2024 Nov 6;10(45):eadr1531. doi: 10.1126/sciadv.adr1531 (PMC11540034; doi:10.1126/sciadv.adr1531)
Supplement: Supplementary file 1 — Sections S1 and S2 Figs. S1 to S6 [file sciadv.adr1531_sm.pdf]

Science Advances
AAAS

Supplementary Materials for

Synaptic-like plasticity in 2D nanofluidic memristor from competitive bicationic transport

Yechan Noh and Alex Smolyanitsky

Corresponding author: Alex Smolyanitsky, alex.smolyanitsky@nist.gov

Sci. Adv. **10**, eadr1531 (2024)
DOI: 10.1126/sciadv.adr1531

This PDF file includes:

Sections S1 and S2
Figs. S1 to S6

S1. Extended data and discussion of the effects of pulse interval, bicationic electrolytes, bias reversal, bias superposition, and membrane material.

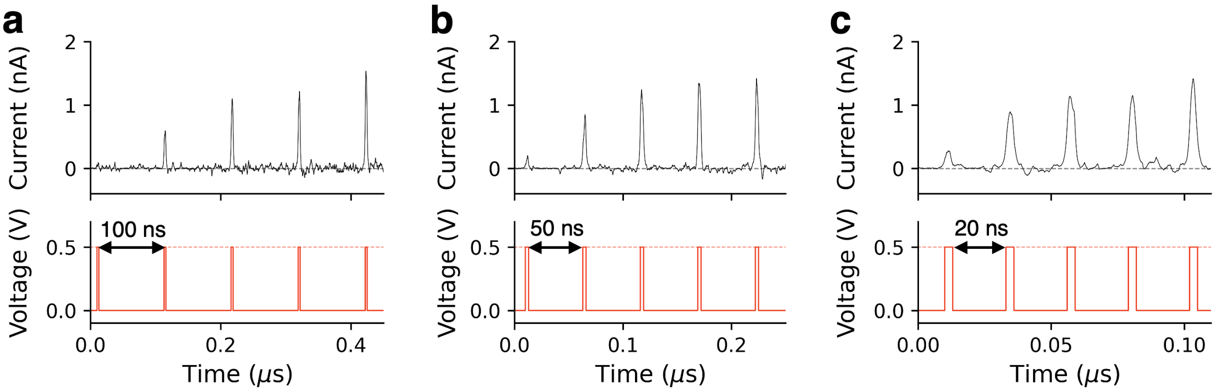

FIG. S1: **Ion current potentiation under voltage spikes applied at different intervals. a.** Δt = 100 ns **b.** Δt = 50 ns**c.** Δt = 20 ns. The pulse height V_s is set to 0.5 V and τ = 5 ns.

Progressive increase (potentiation) of ion current in response to a series of five successive voltage pulses, applied at the intervals of 20 ns, 50 ns, and 100 ns is shown in Fig. S1. The results show that the hBN membrane with B_3N vacancies exhibits consistent current potentiation in all cases.

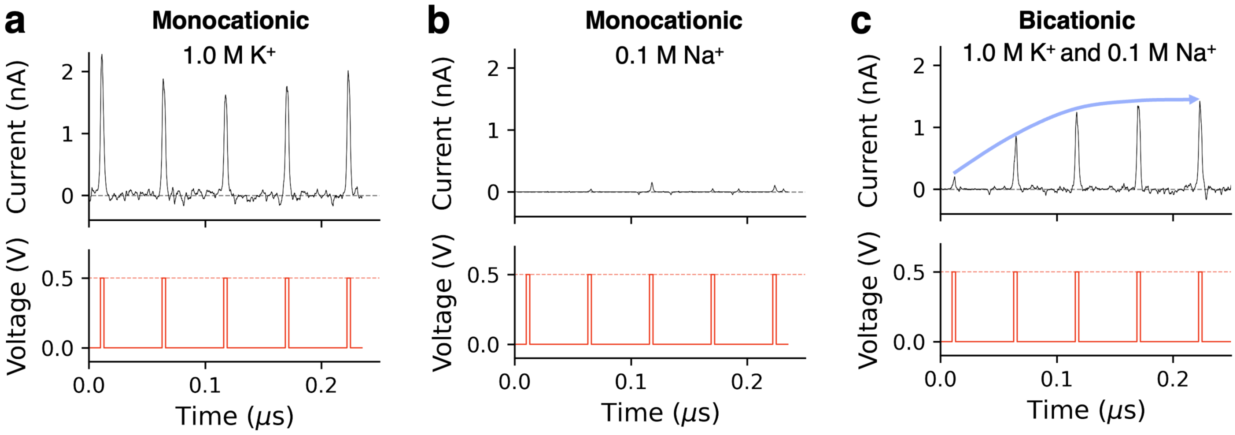

FIG. S2: **Monocationic system *vs* bicationic system.** Ion current induced by five voltage pulses for monocationic systems with **a.** 1.0 M KCl and **b.** 0.1 M NaCl, and bicationic systems with **c.** 1.0 M KCl and 0.1 M NaCl. The pulse height V_s is set to 0.5 V and τ = 5 ns with 50 ns pulse interval.

Fig. S2 shows the important role of bicationic electrolyte in the synapic-like potentiation. When only K^+ ions are present, a uniform response of ion current pulses is exhibited

under successive voltage spikes, as shown in Fig. S2a. In this case, the conductive state of the membrane remains un-modulated, as all pores stay activated in the absence of ions blocking and unblocking pores (Na^+, in this case) with significant time delays. Conversely, in the monocationic system featuring only Na^+ ions, the simulated ion currents are negligible. It is worth noting that beyond a certain number of pulses, the difference between the analytical asymptotic value of ion conductance and that obtained from individual MD simulations would indeed be a number rooted in the stochastic component of the individual simulations. For an experimental system, however, this stochastic discrepancy is expected to be significantly lower.

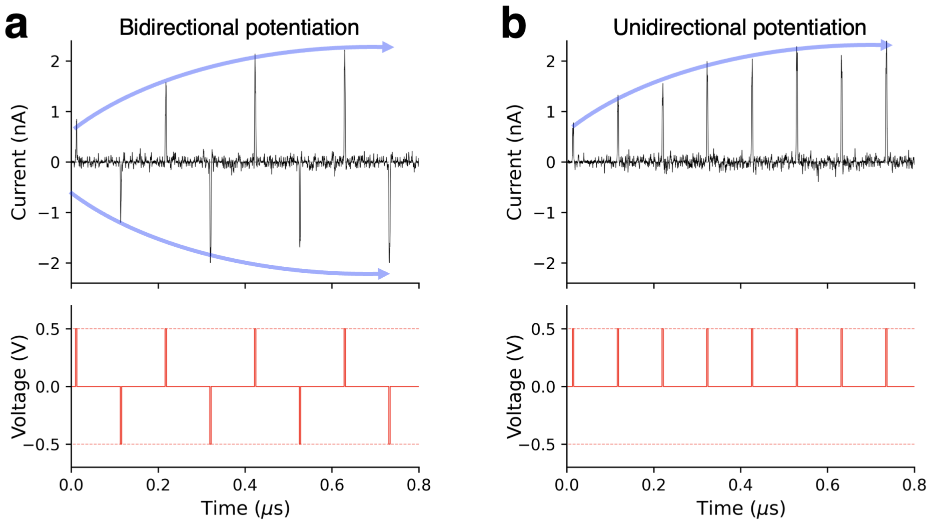

FIG. S3: **Bidirectional current potentiation.** A sequence of voltage pulses of **a.** alternating and **b.** single polarity. The pulse height V_s is set to 0.5 V and $\tau = 3$ ns with 50 ns pulse interval.

As suggested in the discussion presented in the main text, this 2D membrane functions as a bidirectional memristor, as depicted in Fig. S3. The figure illustrates that the ion current is potentiated regardless of bias voltage polarity, as expected for the presented Z-symmetric configuration.

As described in the main text, both the peak current (I_{peak}) and the change in memristive state (Δw) have a nonlinear dependence on the applied voltage magnitude. Thus, as shown in Fig. S4, the resulting current peak is significantly higher when two voltage spikes occur simultaneously, compared to a case of two voltage spikes occurring 10 ns apart.

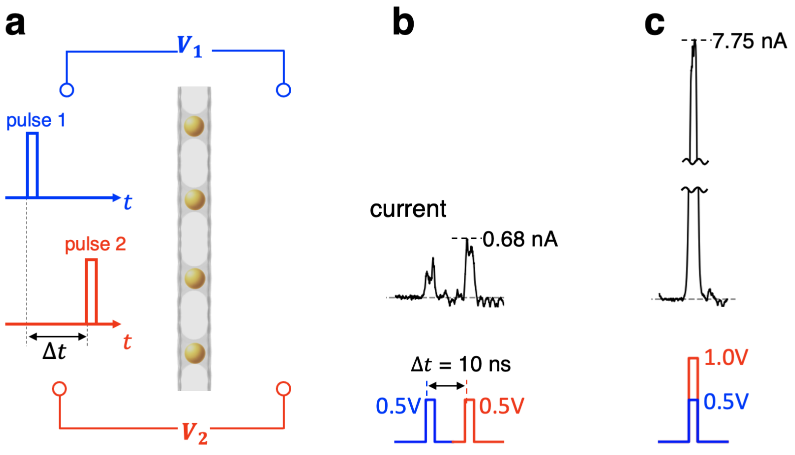

FIG. S4: **Spiking time-dependent current. a.** An illustration of two external voltage sources attached in parallel, generating pulses with a timing difference of Δt. Ion current for the spiking time difference of **b.** Δt = 10 ns and **c.** Δt =0, with a superimposed voltage.

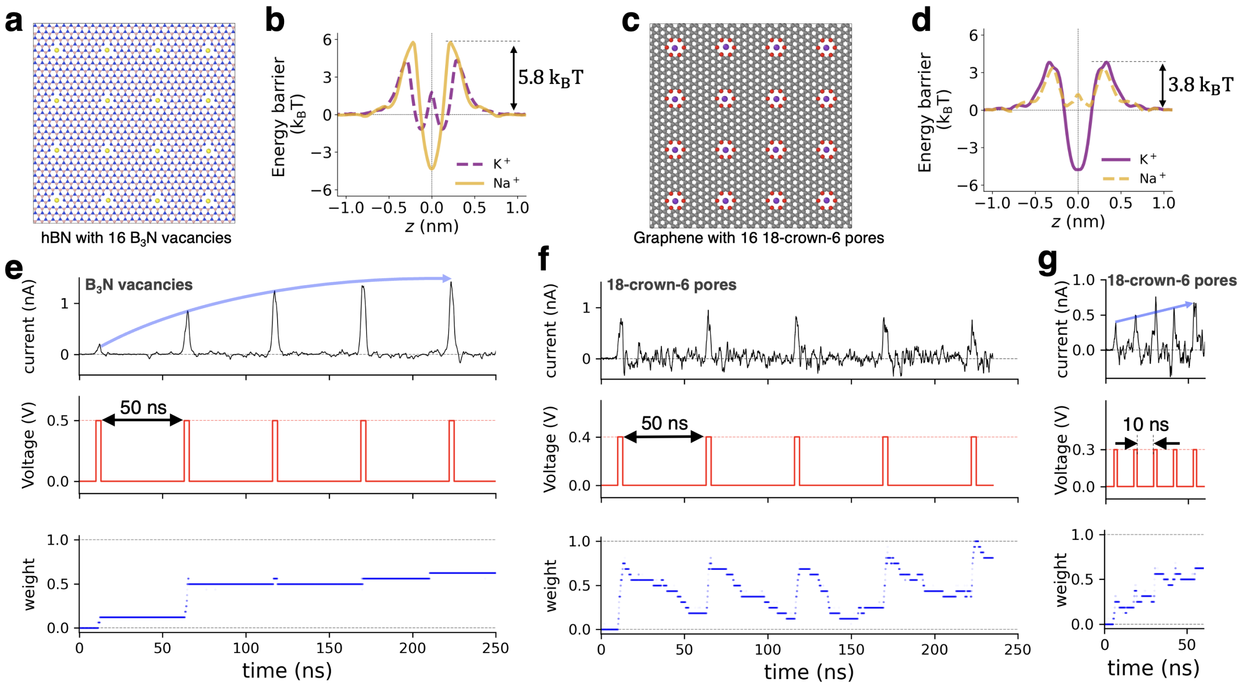

FIG. S5: **Monolayer hBN with 16 B_3N vacancies *vs* Monolayer graphene with 16 18-crown-6 pores. a.** A top view of the hBN sheet with 16 B_3N vacancies. The colors of the atoms are as follows: Nitrogen - blue; Boron - pink; Sodium ion - yellow. **b.** Energy barrier along the transport coordinate across B_3N vacancy. **c.** A top view of the graphene sheet with 16 18-crown-6 pores. The colors of the atoms are as follows: Carbon - gray; Oxygen - red; Potassium ion - purple. **d.** Energy barrier along the transport coordinate across the 18-crown-6 pore. Ion current, voltage, and weight as a function of time for **e.** the hBN sheet with a 50 ns time interval between pulses, **f.** the graphene sheet with a time interval of 50 ns, and **g.** the graphene sheet with a time interval of 10 ns.

We compared the ion current dynamics exhibited by two distinct 2D nanofluidic memris-

tors when subjected to a series of five consecutive voltage pulses. The first system consists of hBN with an array of B_3N defects, as described in the primary discussion of our manuscript and shown in Fig. S5a. The second system is a single layer of graphene featuring an array of 16 18-crown-6 pores, as shown in Fig. S5d. This membrane is known to exhibit memristive effects through the same mechanism described in the main text, except with the roles of Na^+ and K^+ ions are reversed, as suggested by the qualitatively opposite energy landscapes shown in Figs. S5b, S5e. Specifically, in the case of the 18-crown-6 pore array, Na^+ serves as the primary charge carrier across the membrane, while K^+ ions act as pore blockers. As observed previously, the hBN membrane with B3N vacancies exhibits potentiation in ion permeability under a 50 ns pulse interval (see Fig. S5c). However, the graphene membrane with 18-crown-6 pores does not show notable potentiation under a 50-ns-long pulse (see Fig. S5f), due to the considerably faster weight decay between potentiating pulses, as supported by a 2 kBT-lower adsorption energy barrier in the case of crown-like pores in graphene. For a 10 ns pulse interval, the 18-crown-6 pore array exhibits slight ion current potentiation (see Fig. S5g). This example illustrates different operational pulse frequencies depending on the material, as well as pore composition and geometry of a 2D nanofluidic memristor.

S2. Adsorption-desorption dynamics of a binary mixture

The model is similar to that discussed in the main text, except here the competition for the adsorption sites (B_3N pores) is included explicitly. For two cation species, the governing system of equations is:

$$\begin{cases} \frac{dN_1}{dt} = -r_{d,1}N_1 + r_{a,1}(N_{tot} - N_1 - N_2), \\ \frac{dN_2}{dt} = -r_{d,2}N_2 + r_{a,2}(N_{tot} - N_1 - N_2) \end{cases} \tag{S1}$$

The constants are defined in a simplified way as $r_{d,i} = f_{d,i} \cosh\left(\frac{q\phi}{2k_BT}\right) \exp\left(-\frac{E_{d,i}}{k_BT}\right)$ and $r_{a,i} = \kappa_i c_i \exp\left(-\frac{E_{a,i}}{k_BT}\right)$, where $i = 1, 2$ refers to Na^+ and K^+, respectively. For simplicity,

we set $f_{d,1} = f_{d,2} = 16.5$ GHz, $\kappa_1 = \kappa_2 = 14.9$ GHz·M^{-1}, while the barrier heights are taken directly from the data in the main Fig. 1b: $E_{d,1} = 10.1k_BT$, $E_{a,1} = 5.8k_BT$, $E_{d,2} = 5.5k_BT$, $E_{a,2} = 4.3k_BT$. Unbiased ($\phi = 0$) time-decays starting at $w_0 = 1$ for several values of KCl concentration and NaCl fixed at 0.1 M are shown in Fig. S6. Both the decay rate and $w_{t\to\infty}$ depend on the KCl concentration and can be estimated analytically. We briefly focus on the latter, which is given by $w_{t\to\infty} = \frac{\lambda_1(\lambda_1+\lambda_2)}{\lambda_1+\lambda_2+\lambda_1\lambda_2}$, where $\lambda_i = r_{d,i}/r_{a,i}$. This expression is identical to the pore occupancy estimates presented in the supplementary section 3 of our earlier work (26). The corresponding $w_{t\to\infty}$ as a function of KCl concentration is shown in the inset of Fig. S6.

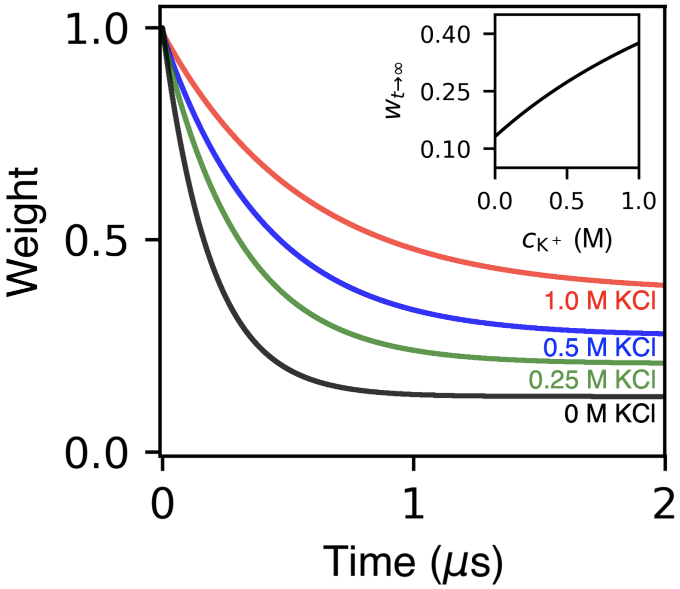

FIG. S6: **Weight decaying over time in systems with a binary salt mixture of varying concentrations of KCl and 0.1 M NaCl.** The inset shows the asymptotic value of w *vs* KCl concentration
